# Spontaneous cerebrospinal fluid rhinorrhoea during pregnancy-case report and review of literature

**DOI:** 10.1515/crpm-2023-0006

**Published:** 2023-04-17

**Authors:** Alma Mackert, Xezal Derin, Parwis Agha-Mir-Salim, Wolfgang Henrich

**Affiliations:** Department of Obstetrics and Gynecology, Charité University of Berlin, Berlin, Germany; Department of Otorhinolaryngology, Head and Neck Surgery, Vivantes Clinic Friedrichshain, Berlin, Germany

**Keywords:** CSF fistula, CSF leakage, idiopathic intracranial hypertension, liquorrhea, pregnancy, rhinorrhoea

## Abstract

**Objectives:**

Rhinoliquorrhea is a condition where cerebrospinal fluid (CSF) leaks due to a liquor fistula formation of traumatic or non-traumatic origin. It can be associated with increased intracranial pressure often due to idiopathic intracranial hypertension (IIH), typically found in young and obese female patients.

**Case presentation:**

A 27-year-old woman, 2 gravida, 1 para, presented with clear rhinorrhoea. After a beta-trace-protein test the diagnosis of CSF leakage was determined. The woman had had a traumatic car accident in 2018 but had never developed clear rhinorrhoea, especially not in her first pregnancy after the accident. Due to stable condition of the mother further diagnostics were postponed until after the birth. An elective caesarean section was performed in 40 + 0 weeks of gestations. The structural bone defect in the posterior wall of the sphenoid sinus was surgically repaired by defect coverage postpartum.

**Conclusions:**

Nasal CSF leakage in pregnancy has previously been described in four other case reports with mostly traumatic etiology. Additionally, IIH is an important diagnosis to keep in mind. So far there are no guidelines or evidence-based recommendations regarding to optimal fistula treatment of pregnant women available. For therapy a prophylactic antibiotic therapy, surgical reconstruction with sealing and a wait-and-see strategy should be considered and discussed.

## Introduction

The medical term Rhinoliquorrhea describes the nasal leakage of cerebrospinal fluid (CSF). These leaks are mostly based on a dural dehiscence of the anterior skull base. The fistula can occur post-traumatically (44 % of cases), postoperatively/iatrogenically (12 %) or spontaneously (28 %). Approximately 16 % of the cases remain of unclear etiology [1].

There are two main diagnostical aspects of a rhinoliquorrhea: proof of CSF and topographic location of the leak.

The proof of CSF is crucial to distinguish against other differential diagnosis such as an allergic or a non-allergic rhinitis. Beta-2-transferrin and beta-trace-protein tests are the most sensitive methods for CSF detection [2]. For location of the leak a Computed Tomography (CT) scan especially in bone window technique is recommended as first-line study to detect a possible bone defect and intracranial air as a secondary sign [3]. Metaanalysis showed that the main localization of the leak is the ethmoid roof (33 %), followed by cribriform plate (28 %), sphenoid sinus (22 %), frontal sinus (8 %) or other sides (9 %) [1]. The symptoms can vary. Lesions of the cribriform plate in the anterior skull base are associated with a posterior or anterior nasal drip. Other possible nonspecific symptoms such as headaches or anosmia can occur [4]. A spontaneous closure is possible but in many cases changing symptoms dependent on the leak activity can be observed [5]. Nevertheless, once a defect is identified, an operative reconstruction should be discussed, because of possible severe complications as a meningitis. The risk for an ascending infection can increase if the nasal CSF fistula persists for over 7 days [6]. The endoscopic intranasal approach using functional techniques (FESS) with repair of the leak using auto-or allografts currently is the standard method. Intracranial surgical repair is to be critically indicated for larger defects or in revision cases [7].

Another possible reason for liquorrhea can be an elevated intracranial pressure. Idiopathic intracranial hypertension (IIH) can occur especially in obese women and lead to a CSF fistula. It is associated with additional symptoms such as headache, transient visual losses, or diplopia [8]. An important diagnostic criterium is an ophthalmological diagnosed papilledema. For pregnant women a IIH prevalence of 5–8 % has been reported [9]. In literature, the context of an elevated intracranial pressure as found in IIH and liquorrhea is already established. The prevalence of definite and presumed IIH in patients with spontaneous CSF leakage was 28 % [10].

## Case presentation

A 27-year-old patient, gravida 2, para 1, was admitted to our clinic for elective caesarean section because of nasal liquorrhea due to a CSF leak.

The patient initially presented at 23 weeks of gestation in an ENT clinic due to repeating rhinorrhoea of a clear liquid for two months. Furthermore, she reported of cephalgia and dizziness. Other symptoms like nausea, neck stiffness or awareness deficits was not observed.

The diagnosis of a nasal CSF leak was proven by several positive beta-trace-protein tests with a secretion/serum ratio of 147 mg/L (reference value >2 mg/L for possible CSF contamination in serum).

The patient had suffered a head trauma following a car accident three years ago. A CT scan directly after the accident had shown no injury or secondary symptoms. Apart from this, her past medical history was inconspicuous except of an adipositas. Her obstetric history consisted of one spontaneous birth without complications. Interestingly, during her first pregnancy in 2018 (after the car accident) she had never shown any symptoms or complaints. She was completely asymptomatic for three years until she developed nasal secretion during the second pregnancy. Usually this is an indication for imaging using CT scans. Because of uncomplicated pregnancy status with only intermittent nasal CSF leakage, it was to be performed postpartum.

For this second pregnancy a caesarean section was suggested to the patient due to the peripartum pressure increase with a risk of fistula enlargement or air embolism (pneumocephalus in the subarachnoid space or ventricular system). We performed the caesarean section without any complications in spinal anaesthesia at 40 + 0 weeks of gestations. She recovered from the procedure without developing neurological symptoms or complaints.

Several weeks after delivery the patient presented at our hospital with severe headaches and dizziness accompanied by subjective gait unsteadiness as well as rhinorhea. The headache slightly worsened when straightening up. For exclusion of a meningitis a CT and MRI scan of the head as well as CSF diagnostic were conducted. There were no signs of infection. Intracranial hypotension and bleeding were also excluded radiologically. A clear bone defect was not found.

Following this incident, an operation was performed. The liquor fistula in the sphenoid sinus was closed using Functional Endoscopic Sinus Surgery (FESS). Due to rhinorrhoea on the first postoperative day, surgical revision was performed by applying TachoSil and fibrin adhesive to the defect localization. For prophylactic purposes Ceftriaxon was applied intravenous during the stationary residence.

In the following days as well as throughout the follow-up appointments no liquorrhea or other complications were detected.

## Discussion

CSF leakage in pregnancy is only rarely discussed in literature. After PubMed research, only four case reports of pregnant women with either idiopathic or manipulation-based CSF leakage are described (Table 1).

**Table 1:** Summary of cases in literature with liquorrhoe during pregnancy.

Age, year	Parity	Gestational age at presentation	Presenting symptoms	Etiology	Therapy	Maternal outcome	Mode of delivery	Reference
30	3G/1p	5	– Cephalgia (forehead) and watery nasal discharge – Rhinorrhoea intensified by valsava maneuver	Nasal polypectomy 9 months ago that caused a defect in cribriform plate with cerebral parenchymal herniation (encephalocele) and liquorrhoe	Defect occlusion by endoscopic transnasal reconstruction	Good recovery	Caesarean re-section	Soltani et al. [11]
34	2G/0p	First trimester	Rhinorrhoea	Endoscopic sinus surgery 11 months ago	Endoscopic repair 3 months postpartum	Unsuccessful surgery	Caesarean section	Schabel et al.
34	–	13	Watery nasal discharge and right-sided hearing loss	Spontaneous defects in ethmoid and mastoid roof	Surgically closed with duraplastie	Good recovery	Caesarean section	Schraven et al. [12]
29	–	Immediately after delivery	Rhinorrhoea associated meningitis	– Reopening of fistula during birth after severe head trauma with a fracture of the skull and paranasal sinus at age of 17 – Ascending infection	–	Postpartum death (6 weeks after delivery)	Vaginal birth	Behrens et al. [13]

Soltani et al. describe a case of a 30-years-old woman in fifth gestational week with CSF leak related rhinorrhoea. Here, a complicated nasal polypectomy with injury of the cribriform plate was performed 9 months before followed by a cerebral parenchymal herniation (encephalocele) and CSF fistula [11].

A similar case is presented by Schabel et al.: A woman complained about rhinorrhoea in first trimester of pregnancy after an endoscopic sinus surgery 11 months ago that caused an iatrogenic defect [14].

Schraven et al. published a case of CSF leakage in a 34-years-old pregnant woman due to spontaneous defects in ethmoid and mastoid roof without any traumatic correlation [12]. Primary elective caesarean sections were performed in all three cases. Another case showed the sequel of CSF leakage: a 29-years-old woman died postpartum because of a meningitis caused by an ascending infection through a CSF fistula. Here, the rhinorrhoea started immediately after vaginal birth. The patient had had a severe head trauma with a fracture of the skull and the paranasal sinuses 12 years before this lethal event. Transient elevated intracranial pressure during birth with accompanying changes in brain tissue during pregnancy was suspected as the cause of the occurrence of the CSF leak [13]. Indeed, the intracranial pressure increases during vaginal birth due to skeletal muscle contractions and not because of uterine contractions [15].

Interestingly, in our case the first pregnancy after the head trauma was asymptomatic without any suspicious nasal secretion. It seems likely that another trigger other than the car accident was present in her second pregnancy. Pérez et al. assert that patients with IIH and CSF fistulas have similar characteristics such as female gender, rather young age and especially high BMI [16]. These characteristics fit for our patient, too, even if she never got the diagnosis or a diagnostic test for papilledema. Since the loss of liquor is also therapeutic against elevated intracranial pressure, the symptoms of IIH are often atypical and therefore the diagnosis can be difficult [10]. There have been cases with CSF leakage where IIH was first diagnosed postoperatively since the defect closure led to increasement of the intracranial pressure [16]. The traumatic accident combined with IIH could in our opinion explain the spontaneous CSF leakage during her pregnancy. The authors of case 3 also suspected IIH as a possible explanation for CSF rhinorrhoea [12]. In our case the radiological images did not present signs of IIH. Since the operation our patient has not developed any symptoms. However, a symptomatic IIH due to pressure increase after defect occlusion can occur anytime.

A third aspect that should be considered is a possible correlation to the SARS CoV 19 pandemic. The patient had a COVID-19-infection before the chronic rhinorrhoea started and reported a harsh nasopharyngeal COVID-19 swab test shortly before the onset of symptoms. Cases of traumatic nasopharyngeal swab testing are described in literature as well [17, 18]. Nevertheless, a nasal CSF leak after a nasal swab is nearly impossible and couldn’t be reasonably determined in our case.

Posttraumatic situations especially after head trauma are common reasons and should be kept in mind, especially if the time between trauma and clinical CSF fistula occurrence is longer than a few months [14].

Finally, physiologic changes during pregnancy in all parts of the body may also impact brain tissue.

In our case the fistula was completely asymptomatic during pregnancy with only rare and intermittent activity. Therefore, there was no clear indication to intervene during the pregnancy. Only in case of a larger defect the surgical reconstruction is indicated. If the skull base is intact and additionally the CSF leakage has stopped spontaneously a wait-and-see strategy is possible.

## Conclusions

CSF leakage in pregnancy is rare and described only in four case reports. There are no guidelines or evidence-based recommendations regarding the optimal fistula treatment of pregnant women as well as the most favourable mode of delivery. Pregnant women with rhinorrhoea should be provided with a prompt beta-trace-protein-test. A detailed anamnesis regarding traumatic events, IIH and endonasal/intracranial surgery is essential. After the diagnosis of CSF leakage, patients should be closely followed up – especially looking out for meningitis symptoms. It should be discussed if a prophylactic antibiotic therapy is necessary. The best prevention of a meningitis with a possible fatal outcome is the surgical closure of the defect even if the need such a procedure should be considered cautiously during pregnancy. In case of complications, a surgical reconstruction should follow immediately. For cases without any complications, a wait-and-see strategy and further diagnostic postpartum seems the best option. An interdisciplinary care for these patients is mandatory.
